# The relationship between lower extremity alignment and Medial Tibial Stress Syndrome among non-professional athletes

**DOI:** 10.1186/1758-2555-1-11

**Published:** 2009-06-11

**Authors:** Golam Reza D Raissi, Afsaneh D Safar Cherati, Kourosh D Mansoori, Mohammad D Razi

**Affiliations:** 1 Iran University of Medical Sciences, Physical Medicine and Rehabilitation Department, Sports Medicine and Orthopedic Department, Tehran, Iran

## Abstract

**Objective:**

To determine the relationship between lower extremity alignment and MTSS amongst non-professional athletes

**Design:**

In a prospective Study, sixty six subjects were evaluated. Bilateral navicular drop test, Q angle, Achilles angle, tibial angle, intermalleolar and intercondylar distance were measured. In addition, runner's height, body mass, history of previous running injury, running experience was recorded. Runners were followed for 17 weeks to determine occurrence of MTSS.

**Results:**

The overall injury rate for MTSS was 19.7%. The MTSS injury rate in girls (22%) was not significantly different from the rate in boys (14.3%). Most MTSS injuries were induced after 60 hours of exercise, which did not differ between boys and girls. There was a significant difference in right and left navicular drop (ND) in athletes with MTSS. MTSS had no significant correlation with other variables including Quadriceps, Tibia and Achilles angles, intercondylar and intermaleolar lengths and lower extremity lengths.

**Limitation:**

All measurements performed in this study were uniplanar and static. The small sample size deemed our main limitation. The accurate assessment of participants with previous history of anterior leg pain for MTSS was another limitation.

**Conclusion:**

Although a significant relationship between navicular drop and MTSS was found in this study; there was not any significant relationship between lower extremity alignment and MTSS in our sample study.

## Introduction

Medial Tibial Stress Syndrome (MTSS) is one of many overuse lower leg injuries found under the overall term of exercise-induced leg pain or shin splints[1]. Stress fractures, MTSS, and chronic compartment syndrome are the three most common forms of exercise "induced leg pain, with MTSS having the highest prevalence[2-4]. MTSS is an exercise-induced, localized pain along the distal two thirds of the posterior-medial tibia and can be a debilitating injury in runners. In studies of recreational runners, MTSS has been reported as the most or second most frequently diagnosed injury and specified 13.2%–17.3% of all running injuries [1-3].

Although the precise pathologic abnormality has not been identified, it appears to involve a stress reaction by the crural fascia (fasciitis) or bone along the posteromedial portion of the tibia most consistently associated with the insertion of soleus, and probably not the periosteum [5].

MTSS can be accurately diagnosed with a clinical history and physical examination [1-4]. Pain can be induced by exercise, last for several hours or days, without dysesthesia and reproduced upon firm palpation of the posterior medial border of the tibia. Imaging investigation like plain radiography, bone scan and MRI should be implemented in order to rule out other conditions such as a stress fracture. However, many studies indicated that some types of imaging have been associated with both false-positive and false-negative results, it has therefore been suggested that if the clinical picture showed MTSS and there is no diagnostic enigma, further images are not necessary [2].

A few studies have reported the injury rate and relationship between lower extremity alignment and MTSS among adolescent runners [6,7]. Bennett et al have studied the relation of pronatory foot type using and MTSS. They mentioned that the combination of sex and navicular drop test measures provides an accurate prediction for development of MTSS [4]. In Plisky et al's study navicular drop has not been considered an appropriate measure to identify runners who may develop MTSS during a cross-country season [8]. In 2007, Reinking et al have studied intrinsic and extrinsic risk factors contributing Exercise-related Leg Pain (ERLP) in collegiate cross-country athletes [9]. The percentage of athletes with history of ERLP associated with running, the incidence of ERLP during one season, factors including years of collegiate running, training distance, and gender associated with ERLP and relationship between foot type and ERLP have been investigated. They concluded that ERLP history and season incidence was common among cross-country athletes and the only risk factor identified for season incidence of ERLP was a history of ERLP [9].

In 2004, Burne 'et al reported a 12-month prospective clinical study on biomechanical and anthropometric risk factors associated with Exertional Medial Tibial Pain (EMTP)[10]. They found that greater internal and external hip range of motion and lower lean calf girth as biomechanical factors were associated with male EMTP; however, the increased incidence of EMTP in women was without any identifiable risk factor [10].

Tweed et al investigated the relationship between functional and static foot posture and medial tibial stress syndrome in distance runners [11]. Variables identified as being significant predictors of medial tibial stress syndrome were the difference between the neutral and relaxed calcaneal stance positions, range of motion of the talocrural joint with the knee extended, early heel lift and abductory twist during gait, and apropulsive gait. They concluded that runners with suspected symptoms of MTSS should be assessed dynamically and statically for abnormal or mistimed pronation[11].

Most of the studies published to date have different results [4,8]. On the other hand, some investigations have done on professional athletes [6,9,11]. Due to possible selected exclusion in persons with MTSS from professional competition, investigation on nonprofessional athletes may determine the effects of potential risk factors more accurately; therefore, we prospectively studied physical education students to determine potential risk factors for the development of MTSS. Considering that the development of MTSS is likely multifactorial, we also examined other risk factors such as gender, running experience, and body mass index (BMI).

## Methods

This prospective study was conducted at Rajaii educational exercise training university in Tehran from September 2006 to January 2007. Our population included 66 nonprofessional athletes (educational exercise students) who were enrolled in track- and- field as a main course. Twenty-one male and 45 female (age 20 ± 1.5 years, BMI 21 ± 2 Kg/m^2 ^and height 1.68 ± .9 m) were studied. Subjects with a history of congenital deformity, surgery and traumatic injuries to either lower extremity in the previous 6 months were excluded. A session was held at university to distribute consent forms and give an overview of the research.

The measures were taken in the first 3 weeks of the semester. At the time of measurement, each subject signed consent form and the examiner completed a demographic data sheet that included name, sex, age, and number of competitive running years. The initial sample consisted of 68 runners, 21 men and 47 women, which provided 136 limbs. Two of runners were eliminated because of injury during the course. Data pertaining to past history of leg pain, injury or fracture, number of hours in physical activity per week, type of physical activity and associated diseases were collected in the questionnaire.

Static lower extremity measurements including intercondylar interval of femur, intermalleolar distance, Quadriceps angle, hip internal rotation angle, hip external rotation angle, tibiofemoral angle, tibial alignment, rear foot alignment or calcaneal (Achilles) angle, navicular drop test and length of lower limb were measured in every lower limb.

All angle measurements were taken using one goniometer and recorded. The measurements were taken 2 times and an average was calculated. On all subjects, the anatomical landmarks were drawn by a nontoxic ballpoint pen and erased after first set of measurements with an alcohol swab. Then several photos were taken from knee and ankle of limbs by a digital ixus 70 Canon camera (with 7.1 mega pixel resolutions). All photos were taken bare foot, in standing position, in specific distance and with constant angle in the same location [9,10], then all photographs were analyzed in Photoshop software (version 7.0) for determination of angles in degrees.

All measurements and physical examinations had been performed by one physician (sport medicine specialist). All subjects were trained during educational track- and- field course and they were running, jumping, marching and circuit training 14 hours per week for 17 weeks. The location of training and surface of field was the same for all subjects and the shoes of all subjects were checked for appropriateness. All shoes were common runner type and no one used insole. After the training period (17 weeks), all subjects were examined for clinical sign and symptoms of MTSS. The diagnostic criteria of MTSS included:

Pain history: pain was induced by exercise and lasted for a few hours or days after exercise, located on the posteromedial border of the tibia, no history of paresthesia or other neurovascular symptoms indicative of other causes of leg pain

Location: Over an area minimum of 5 cm, along the posteromedial border of the tibia.

Palpation: Palpation of the posteromedial border of the tibia produced pain that was diffuse in nature and confined to the posteriomedial border of the tibia [2,12].

### Ethics

A session was held at university to inform the subjects. At the time of measurement, each subject signed a consent form. All photos were taken only from Lower extremity. All data and photos were confidential and the results were reported as general overview.

### Measurement protocol

Lower extremity measures were chosen from common standard clinical measures of foot, knee and leg posture. Height was measured by stadiometer, to 0.5 cm, and weight by electronic scale, to 0.1 kg [4,10,13,14].

### Hip range of motion

The hip and knee were flexed a 90° in the sitting position; rotated internally and externally to a firm end feel and the angles relative to the initial position were measured in degrees.

### Intercondylar and intermalleolar interval

With the subject standing upright; with closed feet held parallel to each other, the intervals were measured using a gauge (accuracy 0.1 mm) for estimation of Knee varus, valgus and ankle eversion and inversion.

### Q angles

The Q angle was measured with the subject's knee and hip in extension, and the quadriceps muscle relaxed. First, the center axis of a long-arm goniometer over the center of the patella was placed. Then, the proximal tibia was palpated and the lower goniometer arm along the patellar tendon to the tibial tubercle aligned. The upper arm of the goniometer was pointed directly at the anterior superior iliac spine (ASIS). The angle measured by the goniometer was the Q angle. The angle was marked by a ballpoint pen, after that the photos were taken and in Photoshop software were analyzed and the angles were determined and compared [13,15].

### Tibiofemoral angle

With the subject standing upright; the center axis of a long-arm goniometer was placed over the center of the patella. The upper arm of the goniometer was pointed on the bisection of femur and lower arm along the bisection of tibia and measuring the degree, estimation of femoral and tibial alignment was possible. Then landmarks were drawn by marker, photos were taken and in Photoshop software were analyzed and the angles were determined and compared [16].

### Rear foot alignment (Achilles or calcaneal angle)

With the subject lying prone, one leg extended and the other leg externally rotated and bent at the knee at approximately 90°, the calcaneus and one third of extended lower leg were marked for rearfoot measurements. A line was drawn bisecting the lower one third of the extended leg and another line was drawn bisecting the calcaneus. Inversion and eversion of the calcaneal position was determined in degrees with a goniometer [17,18].

### Tibial alignment

Each leg was measured in standing position. The medial and lateral aspects of the tibia were palpated, and then the medial-lateral bisection was determined. Tibial alignment was measured with a goniometer marked in degree [10,16]. The photos were taken and in Photoshop software was used for the analysis and the angles were determined and compared. Tibial alignment and Q angle were evaluated according to results taken from Photoshop analysis. Other measures were taken from physical examination.

### Navicular drop test

Navicular drop was measured in standing position based on the description by Sell et al [19] and Picciano et al [20]. The navicular tuberosity was palpated at its greatest prominence and marked with a nontoxic marker. In bilateral standing, the subject was placed in subtalar joint neutral. The distance between the floor and the navicular mark was measured in millimeters with goniometer. The subject then stood in unilateral stance, and the examiner again measured the distance (mm) between the floor and the navicular mark. The difference between the navicular height in bilateral and unilateral standing was recorded as the navicular drop [17].

### Leg Length discrepancy

Each leg length was measured using a tape measure (nearest 0.5 cm), in the supine position, from the anterior superior iliac spine to the most prominent aspect of the medial malleolus [10,21].

### Intratest reliability

A pilot study was done to determine the intratester reliability of measurements (kappa = 0.75). Using the procedures described above, 20 limbs from 15 subjects were measured 3 times during 1 week. To eliminate subject identification, random assignment of subject order was performed by computer and measurers were blinded to the subjects.

### Statistical analysis

Descriptive statistics including mean, st. error of mean, median and std. deviation were calculated for runners' characteristics.

Data were typed and analyzed in software of SPSS version 15. For comparing continuous normally distributed data, T-test was performed; and in the case of determining the association between two categorical data, Chi-square test was run.

The Odds ratio & 95% CI were conducted for precision, determination of the effect size and clinical degree of significance.

## Results

Baseline characteristics of the study samples are shown in Table 1. Majority of the participants were female (68%). The runners' average (± SD) age, body mass, and height were 20 ± 1.5 years, 21 ± 2 Kg/m^2 ^and 1.68 ± .9 m, respectively.

**Table 1:** Baseline characteristics of the study samples

**Variables**	**Gender**	**Frequency%**	**Mean ± SD**
Age (years)	Total	66(100%)	20.0 ± 1.5
	Male	21(32%)	20.8 ± 1.7
	Female	45(68%)	20.2 ± 1.3

Height(m)	Total	66(100%)	1.68 ± .9
	Male	21(32%)	1.80 ± 0.5
	Female	45(68%)	1.65 ± 0.4

BMI(Kg/m^2^)	Total	66(100%)	21 ± 2
	Male	21(32%)	21 ± 1.7
	Female	45(68%)	21 ± 1.7

Positive history of Professional activity	Total	26(39.4%)	
	Male	11(42%)	
	Female	15(58%)	

Duration of Professional activity	Total	26(39.4%)	3 ± 2.7
	Male	11(42%)	5 ± 2.9
	Female	15(58%)	2 ± 2.1

Past history of pain	Total	6(9%)	
	Male	2(33%)	
	Female	4(67%)	

Anterior Location of previous pain	Total	4(6.1%)	
	Male	0%	
	Female	4(100%)	

Length of weekly physical activity	Total	66(100%)	13 ± 1.9
	Male	21(32%)	14 ± 2.3
	Female	45(68%)	12.5 ± 1.2

During the 17-week of running program, 13 runners (19.7%) incurred MTSS and 3(4.5%) reported pain in anterior of tibia. The participants developed MTSS bilaterally. The MTSS injury rate in girls (10/45)22% was not significantly different from the rate in boys (3/21)14.3% (Table 2). Most MTSS injuries incurred approximately after 60 hours of training, average of 64 ± 12 hours which did not differ between boys and girls. All of the previous running pain in anterior leg were reported by girls (4/45) 8.8%.

**Table 2:** Clinical findings of the study sample

**Variables**	**Gender**	**Frequency%**	**Mean ± SD**
Navicular Drop (mm) (Right and Left)	Total	66(100%)	5.30 ± 1.2
	Male	21(32%)	5.30 ± 1.5
	Female	45(68%)	5.20 ± 1.7

Medial tibial stress Syndrome (MTSS)	Total	13(19.7%)	
	Male	3(14.3%)	
	Female	10(22%)	

Duration of MTSS injury (hours)	Total	13(19.7%)	64 ± 12
	Male	3(14.3%)	63 ± 15
	Female	10(22%)	64 ± 17

Runners who reported a previous running pain and injury were over 2 times more likely to incur an MTSS, but the association was not statistically significant. Odds ratio & 95% CI were used to establish if the descriptive data could accurately predict the development of MTSS (odds ratio [OR], 2.18; 95% CI: 0.7, 6.4), with similar reports among girls and boys. The small size of study may account for lack of this association.

We found larger navicular drop among participants with MTSS. There was a significant difference in Right ND (6 versus 5.1 mm) (P < 0.05) and Left ND (6.5 versus 4.9 mm) (p < 0.05) among participants with MTSS and those without MTSS (Table 3). Other variables (Q, Tibia and Achilles angles, intercondylar and intermalleolar lengths and lower extremity lengths) did not show a significant relationship with MTSS (Table 4).

**Table 3:** Comparing measured navicular drop in MTSS and NO MTSS subjects

	**Outcome**	**N**	**Mean**	**SD**	**P-Value**	**df**	**t**
Right Navicular Drop test	MTSS	13	6.08	1.553			
					0.027	63	31.546
	NO	53	5.14	1.250			
	MTSS						
Left Navicular Drop test	MTSS	13	6.46	2.184			
					0.034	63	23.681
	NO	53	5.00	1.581			
	MTSS						

**Table 4:** Comparing measured angles (Q, Achille, Tibia) and measured distances (Intercondylar and intermaleolar) in MTSS and NO MTSS subjects

**Angles and distances**	**Outcome**	**N**	**Mean**	**SD**	**P-Value**	**df**	**t**
right Q angle	MTSS	13	11.77	1.536	N.S	63	41.195
	NO MTSS	53	12.08	2.510			
left Q angle	MTSS	13	13.00	1.472	N.S	63	38.23
	NO MTSS	53	12.28	2.885			
right tibia angle	MTSS	13	84.231	2.5614	N.S	63	246.887
	NO MTSS	53	83.945	2.7504			
left tibia angle	MTSS	13	84.515	2.4090	N.S	63	219.512
	NO MTSS	53	83.211	3.1863			
right Achille angle	MTSS	13	8.77	2.587	N.S	63	29.434
	NO MTSS	53	8.96	2.369			
left Achille angle	MTSS	13	8.23	2.682	N.S	63	28.130
	NO MTSS	53	9.46	2.584			
Intercondylar distance of femore	MTSS	13	19.23	16.053	N.S	63	8.253
	NO MTSS	53	13.77	13.929			
Intermaleolar distance	MTSS	13	8.23	10.158	N.S	63	6.610
	NO MTSS	53	5.79	7.129			

## Discussion

In this study the relationship between MTSS and ND was significant, in accordance with Bennett study [4]. Average navicular drop has been reported from 3.0 to 9.5 mm in healthy subjects with the average navicular drop observed in runners with MTSS in this study was 6.8 to 8.9 mm. The mean (± SD) values for right and left navicular drop in our study (5.14 ± 1.2 and 5 ± 1.5 mm for noninjured runners and 6.08 ± 1.553 and 6.46 ± 2.1 mm for runners who developed MTSS, respectively) were not generally higher than previously reported values.

However this finding differs from the study conducted by Plisky et al, who found no association between navicular drop and MTSS in competitive adolescent runners [8]. The discrepancy in findings, may be due to differences in the specific measurement technique [2,4,17]. The Plisky study used the method assessing navicular drop advocated by Menz and others, whereas we used the method described by Sell and Picciano [19,20].

Our incidence estimates of MTSS for the overall sample (19.7%) and girls (22.2%) were not similar to Bennett et al, with incidence estimates of 12% and 19.1%, respectively. The frequency of MTSS assessed in our study was not significantly different between girls and boys (22.2% versus 14.3%). While others have suggested the higher incidence of injury in women rather than men [2,4,10,22], we did not find this issue in our study, however, the frequency of sport activity was nearly the same in both gender. We did not find apparent relationship between lower extremity alignment and MTSS that was consistent with some similar studies [23,24].

Likewise, no significant difference in the rearfoot or calcaneal angle was found between the injured and noninjured groups, contrary to the findings of Viitasalo and Kvist [25].

Considering that eversion is the frontal plane component of subtalar pronation, we expected to find a significant difference between the injured and noninjured groups in rearfoot or calcaneal angle like we found in the navicular drop test. The apparent conflict between the navicular drop test results and the rearfoot or calcaneal angle results may be explained by our use of uniplanar measurements of a triplanar motion (static assessment of a dynamic issue) [13,26].

Moreover, excessive pronation is connected with overused problems of lower leg, in the view of James et al [27] it is a compensatory motion secondary to anatomical misalignment of the heel-foot or leg-foot.

The increased pronation of the subtalar joint may be due to forefoot varus deformity, a rear foot varus deformity, functional equinus and tibial varum. As a conclusion, they pointed out that no single anatomical variation correlated with any specific diagnosis [26]. Therefore, the discrepancy between the result of ND and calcaneal angle in our study may be due to not considering forefoot varus deformity measurement.

According to Tiberio, an increased Q-angle, excessive foot pronation, increased femoral anteversion, and weaker quadriceps together may cause leg pain [28]. In our study, there was no significant difference between Q-angle in MTSS group versus uninjured, so Q-angle may not have a role in contributing MTSS.

We did not find an apparent difference between the injured and noninjured limbs in tibial alignment, comparable to Bennett study [4], but it was in conflict with Tomaros' research [29]. In this study the difference in tibiofibular varum between the injured and uninjured extremities may be one example of an asymmetry that can cause unilateral overuse symptoms [29].

Although runners with MTSS had a little higher BMI, the difference was not significant. So other studies in military recruits had mentioned that BMI remains an equivocal risk factor for any type of lower extremity injury and in the study by Plisky higher BMI was associated with risk of MTSS in runners. In our study, subjects were divided into higher and lower subgroups based on their BMI (> or = 21 and < 21, respectively), then correlational analysis were performed. However, no relationship was found between MTSS and BMI of the participants (Odds ratio [OR] = 0.79; 95%CI: 0.2, 2.68).

Runners who reported a previous running injury were over 2 times more likely to incur MTSS, but the association was not statistically significant (odds ratio [OR], 2.18; 95% CI: 0.7, 6.4) with similar reports among girls and boys. Other researchers have reported an association between prior injury and current injury [5,9,30]. As the small number of MTSS cases observed likely accounted for the statistical finding, it should be suggested that prior injury may play a role in the occurrence of MTSS and warrants further investigation.

In our study some of the subjects had a previous history of anterior leg pain; meanwhile the students were excluded if they had a history of lower limb injury during the previous six months. Hence, there may have been subjects who had an older or less recent history of pain, but did not develop MTSS in the study period. It should be considered as a reason why we may have not found an association.

The main limitation of this study is the small sample size that reduced the power of our analysis, so further studies in larger groups are required.

This research is a prospective study in athletic population and we have highlighted the pronated foot type as a significant risk factor for MTSS. Likewise, application of precise methods to quantify the structural characteristic of lower extremity in this study, made us possible to better define the alignment that may or may not contribute to injury.

It is important to prospectively identify modifiable risk factors so that strategies may be adapted to minimize injury occurrence. If risk for injury can be identified prior to the training season using navicular drop test normalized to foot length, interventions such as custom orthotics or shoe wear prescription may be recommended to the runner to help minimize potential injuries and time lost from sport participation [31]. Prevention methods that should be considered are, shock-absorbent insoles, foam heel pads, Achilles tendon stretching, footwear, and graduated running programs [31,32].

## Conclusion

A significant relationship was found between navicular drop and MTSS in this study. But we did not find any relationship between lower extremity alignment and MTSS. MTSS had no significant correlation with other variables including Q, Tibia and Achilles angles, intercondylar and intermaleolar lengths and lower extremity lengths.

## Competing interests

The authors declare that they have no competing interests.

## Authors' contributions

GRR conceived the main idea, participated in sequence alignment, design of study, statistical analysis and its revision and coordination. ASC drafted the manuscript, participated in data collection and examinations, performed the statistical analysis and carried out the revisions. KM participated in sequence alignment, design of study, statistical analysis and its coordination. MR participated in sequence alignment, design of study and its revision.

## References

[B1] Beck BR (1998). Tibial stress injuries: an aetiological review for the purposes of guiding management. Sports Med.

[B2] Yates B, White S (2004). The incidence and risk factors in the development of Medial Tibial Stress Syndrome among Naval recruits. Am J Sports Med.

[B3] Epperly, Fields K, Oconner F, wilder, R (2001). Epidemiology of running injuries. Textbook of Running Medicine.

[B4] Bennett JE, Reinking MF, Plumer B, Pentel A (2001). Factors contributing to the development of Medial Tibial Stress Syndrome in high school runners. J Orthop Sports Phys Ther.

[B5] Fredericson M, Bergman AG, Hoffman KL (1995). Tibial stress reactions in runners: Correlation of clinical symptoms and scintigraphy with a new magnetic resonance imaging grading system. Am J Sports Med.

[B6] Wen DY (2007). Risk factors for overuse injuries in runners. Curr Sports Med Rep.

[B7] Hubbard TJ, Carpenter EM, Cordova ML (2009). Contributing factors to medial tibial stress syndrome: a prospective investigation. Med Sci Sports Exerc.

[B8] Plisky MS, Rauh MG, Heidersheit B (2007). Medial tibial stress syndrome in high school cross-country runners: incidence and risk factors. J Orthop Sport Phys Ther.

[B9] Reinking MF, Austin TM, Hayes AM (2007). Exercise-related leg pain in collegiate cross-country athletes: extrinsic and intrinsic risk factors. J Orthop Sports Phys Ther.

[B10] Burne SG, Khan KM, Bandeth BP (2004). Risk factor associated with exertional medial tibial pain: a 12 month prospective clinical study. Br J Sports Med.

[B11] Tweed JL, Campbell JA, Avil SJ (2008). Biomechanical risk factors in the development of medial tibial stress syndrome in distance runners. J Am Podiatr Med Assoc.

[B12] Batt ME, Ugalde V, Anderson MW, Shelton DK (1998). A prospective controlled study of diagnostic imaging for acute shin splints. Med Sci Sports Exerc.

[B13] Kaufman KR, Bordine SK (1999). The effect of foot structure and range of motion on musculoskeletal overuse injuries. Am J Sports Med.

[B14] Cheng JCY, Chan PS, Chiang SC (1991). Angular and Rotational Profile of the lower limb in 2,630 Chinese children. J Pediatr Orthop.

[B15] Krivickas LS (1997). Anatomical factors associated with overuse sports injuries. Sports Med.

[B16] Elveru RA, Rothstein JM (1988). Methods for taking subtalar joint measurements. A clinical report. Phys Ther.

[B17] Buchanan KR, Davis J (2005). The relationship between forefoot midfoot, and rearfoot static alignment in pain-free individuals. J Orthop Sports Phys Ther.

[B18] Redmond A (2004). Two feet or one person? (Editorial). The Foot.

[B19] Sell KE, Verity TM, Worrell TW, Pease BJ, Wigglesworth J (1994). Two measurement techniques for assessing subtalar joint position: a reliability study. J Orthop Sports Phys Ther.

[B20] Picciano AM, Rowlands MS, Worrell T (1993). Reliability of open and closed kinematic chain subtalar joint neutral positionsand navicular drop test. Orthop Sports Phy Ther.

[B21] Ekstrand J, Wiktorsson M, Oberg B, Gillquist J (1982). Lower extremity goniometric measurements. Arch Phys Med Rehabil.

[B22] Soderman K, Alfredson H, Pietila T (2001). Risk factors for leg injuries in female soccer players: a prospective investigation during one out-door season. Knee Surg Sports Traumatol Arthrosc.

[B23] Griffin LY, Shangold NM, Mirkin G (1994). Orthopedic concerns. Women and exercise: physiology and sports medicine.

[B24] Beck BR, D Louis R (1994). Medial Tibial Stress Syndrome. J of Bone and Joint Surgery.

[B25] Viitasalo JT, Kvist M (1983). Some biomechanical aspects of the foot and ankle in athletes with or without shin splint. Amer J Sport Med.

[B26] Willems TM, De Clercq D (2006). A prospective study of gait related risk factors for Exercise-related Lower Leg Pain. Gait & Posture.

[B27] James SL, Bates BT, Osternig LR (1978). Injuries to runners. Am J Sports Med.

[B28] Tiberio D (1988). Pathomechanics of structural foot deformities. Phys Ther.

[B29] Tomaro J (1995). Measurement of tibiofibular varum in subjects with unilateral overuse symptoms. J Orthop Sports Phys Ther.

[B30] Stevenson MR, Hamer P, Finch CF (2000). Sport age, and sex specific incidence of sports injuries in Western Australia. Br J Sports Med.

[B31] Thacker SB, Gilchrist J, Stroup OF, Kimsey CD (2002). The prevention of shin splints in sports: a systemic review of literature. Med Sci Sport Exerc.

[B32] Craig DI (2008). Medial Tibial Stress Syndrome: evidence-based preventions. J Athl Train.

